# Looked at Life from Both Sides Now

**DOI:** 10.3390/life4040887

**Published:** 2014-12-11

**Authors:** Jillian E. Smith, Allisandra K. Mowles, Anil K. Mehta, David G. Lynn

**Affiliations:** Department of Chemistry and Biology, Emory University, 1515 Dickey Drive, Atlanta, GA 30322, USA; E-Mails: jillian.smith@emory.edu (J.E.S.); amowles@emory.edu (A.K.Mo.); akmehta@emory.edu (A.K.Me.)

**Keywords:** dynamic chemical networks, digital and analog information, prions, amyloid, chemical and biomolecular evolution, molecular mutualisms

## Abstract

As the molecular top–down causality emerging through comparative genomics is combined with the bottom–up dynamic chemical networks of biochemistry, the molecular symbiotic relationships driving growth of the tree of life becomes strikingly apparent. These symbioses can be mutualistic or parasitic across many levels, but most foundational is the complex and intricate mutualism of nucleic acids and proteins known as the central dogma of biological information flow. This unification of digital and analog molecular information within a common chemical network enables processing of the vast amounts of information necessary for cellular life. Here we consider the molecular information pathways of these dynamic biopolymer networks from the perspective of their evolution and use that perspective to inform and constrain pathways for the construction of mutualistic polymers.

## 1. From Up and Down, and Still Somehow

Life may best be understood as information on a nanoscale [1]. The sequences of DNA and the vast repertoire of catalytic and structural forms of proteins constitute a dynamic evolving network easily seen in the top–down causality of the tree of life. Most remarkable is the information flow achieved with two biopolymers, so universal as to be termed the Central Dogma of biology [2], locked in mutualistic synergy. Hydrogen-bond donor/acceptor pairs bias the folding pathways of nucleic acids to optimally store “digital-like” information in the genome. In contrast, protein folding is context dependent, coupling “analog-like” conformational distributions with inputs from the changing environments that drive evolution. Coordinating these analog and digital forms of information has been heralded as the Darwinian Threshold of cellular life [3], and this entire issue of *Life* strives to review our understanding of how such mutualistic networks could have emerged as a process for biogenesis.

Early experiments [4] showed that amino acids and peptides are readily accessible from simple gaseous precursors, providing a source for the building blocks of biogenesis. The discovery that nucleic acids can serve as catalysts, ribozymes [5,6] and deoxyribozymes [7], proved that these polymers could carry both analog and digital functions, and gave substance to an RNA World hypothesis for early life [8]. Genome sequencing from across all three domains of life and the resulting top–down understanding of the progressive growth of molecular information over time was traced back to the critical threshold for cellular life as the emergence of the ribosome [3]. This organelle, the digital-to-analog converter of the Central Dogma, is itself a supramolecular RNA/protein co-assembly [9] composed of integrated biopolymers that emerged simultaneously [10,11]. Today messenger RNA, the intermediate carrier of genetic information, serves in mutualistic synergy with proteins at every stage of the process. From mRNA-binding proteins during transcription to nuclear membrane transport, from being spliced, capped and polyadenylated by ribonucleoprotein complexes (RNPs) to the mRNA-protein complexes necessary for translation in the ribosome, this positive reciprocal relationship between nucleic acids and proteins within dynamic networks provides the foundation for genomic biological information flow [12].

**Figure 1:**
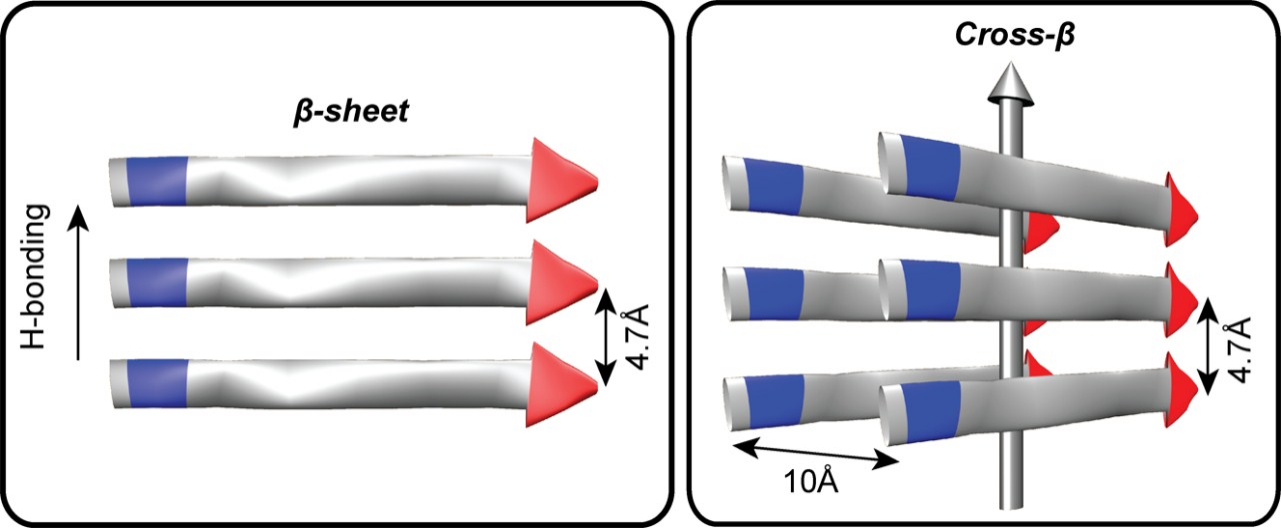
The architectural core of prions and amyloids. Fibers are defined both by the length of the H-bonded β-strands and β-sheet stacking, or lamination. The distance between two H-bonded peptides in a β-sheet is 4.7Å and the approximate distance between laminated β-sheets is 10Å. The amino (*N*) terminus is colored blue and the carboxy (*C*) terminus is colored red. The vertical grey arrow indicates the H-bonding direction and the typical direction of the amyloid fiber long axis.

The origins of this protein/nucleic acid biopolymer mutualism can now be reconsidered in light of the discovery of protein-only infectious agents known as prions [13]. Prion proteins undergo Darwinian-like selection and propagation from a clonal population of diverse assembled phases existing within a cellular matrix. While the basis for such selection in disease remains unknown, the process achieves gain-of-function phenotypes from folding and assembly of cross-β forms [14,15,16] (Figure 1). These scaffolds also serve as adhesives in bacterial biofilms [17,18], as heritable elements in fungi [19], and in one, the Het-s amyloid, the assembly mediates self/non-self recognition to control incompatible fusions [20]. Increasingly beneficial functions of amyloids are being found in multicellular organisms, including the protective egg envelope of *Austrofundulus Limnaeus* embryos [21], barnacle adhesives [22], weevil cocoons [23], and *Crysopa flava* (lace-wing fly) silks [15]. Orb2 amyloid oligomers contribute to long-term memory of fruit flies [24] and the Pmel17 amyloid fibers appear to play a role in directing the polymerization of the skin pigment polymer melanin [25]. These current functions of amyloid highlight the evolutionary potential of assembled protein phases and underscore the possibility of independently evolving nucleic acid and protein biopolymers, all of which could enrich the obligate mutualisms seen in biological information networks.

As Joni Mitchell sang over 40 years ago, “I have looked at life from both sides now…, and still somehow…, I don’t know life at all”. Since that time, the structural and functional analysis of both sides of biological information flow have revealed the richly dynamic far-from-equilibrium networks of nucleic acids and peptides that enable biopolymer evolution. Here we review some of this growth in the context of chemical evolution, looking at life from the above and below that is today informing the potential for the sustained emergence of function in a wider array of dynamic chemical networks.

## 2. Biopolymer Diversity

Discrete geometries of hydrogen-bonding donor and acceptor pairs direct genomic information, achieving “digital-like” coding fidelity. In this case, functional diversity is accumulated through mutations within the nucleic acid polymer sequence that is expressed in phenotypic variations mapped to the tree of life. The iconic right-handed B-DNA double helix [26,27] can adopt other antiparallel double helix conformations, including A-DNA, Z-DNA and A-RNA, and their biological roles are still being explored [28,29,30]. Bulges, hairpins, and cruciform conformations further contribute to the structural diversity and extend the possible functions of this scaffold [31,32]. The ability of nucleic acids to fold into polymorphic non-canonical structures in response to metal coordination (G-quadruplexes) and pH (*i*-motif) [33,34,35], and the growing evidence for these structures existing *in vivo* [36,37], have continued to expand the functional possibilities [38,39]. The extension to new nucleic acid catalysts [5,6,7] through *in vitro* directed evolution is reviewed in this issue of *Life* (Muller) and elsewhere [40,41,42,43]. Clearly the nucleic acid scaffold has remarkable functional potential for both information storage and processing.

Proteins, with an even greater diversity in side chain functionality, are acutely sensitive to amino acid substitutions and are environmentally responsive in their folding dynamics. The polyamide backbone can access α-helices, β-sheets, and turns directed by non-covalent interactions ranging from van der Waals, hydrophobic effects, aromatic stacking, π-cation interactions, hydrogen-bonding and electrostatic interactions. All these factors influence secondary and tertiary peptide conformations in context-dependent ways [44,45] and provide remarkable diversity that still defies 3° and 4° structural predictions [44,46].

The growing recognition of the prevalence of protein misfolding diseases and prion infections has focused greater attention on defining higher order 4° structures and protein phases [47,48,49]. A central feature of all known prion and amyloid fibers is the range of accessible paracrystalline cross-β architectures [50,51]. Amyloid fiber cross-section is defined both by the length of the H-bonded β-strand and side-chain interactions that stabilize sheet stacking, or lamination (Figure 1). These fiber ends template the addition of individual peptides, reducing the diverse conformational space any peptide can sample to a single state. Unlike information storage in nucleic acid duplexes, the template cross-β protein strand transmits conformational information to the incoming strand, which then serves as the template for further propagation to the next incoming strand. In prions, specific morphological forms of the peptide cross-β architecture are selected for and propagated from clonal ensembles of diverse amyloid templates [52,53,54,55].

**Figure 2:**
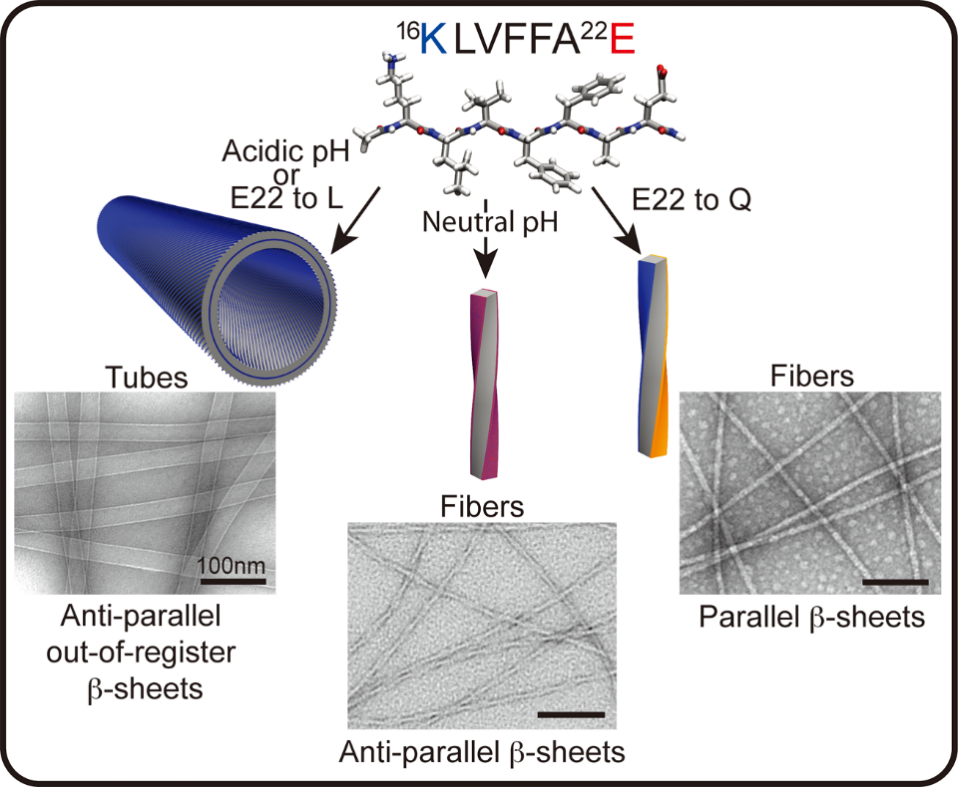
Aβ [16,17,18,19,20,21,22] peptide assembles into distinct morphologies depending on sequence and conditions. In the cartoons above, blue represents the positively charged lysine (K), red the negatively charged glutamate (E), and orange the uncharged glutamine (Q). Two of the four faces of the fiber in the anti-parallel β-sheets present lysine and glutamate residues on the surface (purple), whereas the fibers with parallel β-sheets have a lysine surface and a glutamine surface. (Unpublished EM images from members of Lynn Lab.)

The diversity of conformational forms accessible to these peptide templates may be most easily explored in studies with simple peptides. The nucleating core of the Aβ peptide associated with Alzheimer’s disease, ^17^LVFF^21^A, can access a range of morphological phases, each responsive to pH, media dialectic, surfaces, solvent composition, and various salts [56,57,58,59]. Despite this diversity, specific conditions have been found that allow for the growth of phases sufficiently homogeneous for structural characterization. At neutral pH, anti-parallel β-sheet fibrils predominate with Aβ [16,17,18,19,20,21,22], Ac-^16^KLVFFA^22^E-NH_2_ (Figure 2). Cross-strand pairing between the positively charged lysine and negatively charged glutamate residues of neighboring H-bonded strands defines an in-register arrangement [59,60]. Protonation of the glutamate at low pH favors a shift of the strands to out-of-register, creating more complementary β-sheet faces that allow the number of sheets to grow (laminate) into ribbons and nanotubes [59,61,62,63]. The Aβ [16,17,18,19,20,21,22] E22L congener, Ac-KLVFFAL-NH_2_, removes this ionic pairing constraint completely and assembles independent of pH as nanotubes with the same out-of-register β-strands [57,59,63,64].

Single molecule experiments have been used to map the phase transitions [65,66] and have begun to reveal intermediate nucleation events during conformational progression [67,68,69]. The simple change of one O for an NH in Ac-KLVFFAQ-NH_2_ initially assembles as antiparallel β-strands, which arise as a kinetic intermediate controlled by charge repulsion in the initial particle phase. A secondary conformational mutation stabilized by glutamine side chain cross-strand pairing directs the nucleation and propagation of the thermodynamically more stable parallel β-strand registry [68]. This clear demonstration of kinetic intermediates expands the diversity of accessible informational forms that may propagate under different environmental conditions, and highlights the progressive nature of peptide nucleation and propagation as a mechanism for the selection of functional information.

The diversity of these peptide assemblies is derived not only from primary sequence, but also from the varied arrangements accessible to the ordered phases. In the simplest forms, even single aromatic amino acids [70] and dipeptides, containing aromatic [71,72,73,74] and aliphatic residues [75,76], can assemble as distinct phases. Aliphatic dipeptides (AI, IA, VV, VI, AV, VA, and IV) assemble into hexagonal prisms to form microporous crystalline materials, zeolites [75,76], and various other complex assemblies [77,78]. Diphenylalanine nanotubes (FF) can react with 2-iminothiolane, in which the subsequent thiolation of the *N*-terminal primary amine induces a morphological shift from nanotubes to spherical closed cages [74]. The opportunity to exploit even the simplest of peptide-assembly surfaces as templates for further chemistry with subsequent feedback control of the assembly certainly expands the potential functional diversity. As the structural understanding of these assemblies continues to grow and new surfaces are designed as templates for post-assembly modification, the functional diversity of these forms will continue to extend their informational potential.

## 3. Functional Assemblies

Genomic information present in the one-dimensional nucleic acid templates, as managed by proteins, produces the functional biopolymers of living dynamic cellular networks. Quite possibly the simplest functional form of this molecular information processing is found in viroids. These single stranded RNAs of only a few hundred nucleotides direct pathogenesis in many plant diseases [79]. Viroids are non-protein coding circular structures that assemble as long rod-like forms with a central conserved region and loops containing complex hydrogen-bonding patterns [80,81,82]. One class of viroids contains a hammerhead ribozyme for self-cleavage during replication cycles [80,83,84], allowing many of their functions to be self-contained and hailed as vestiges of an RNA World [85]. However, viroids are informational templates and depend on the host’s cellular network for functional replication.

The paracrystalline cross-β peptide assemblies of the infectious prions are quite similar [50]. Like viroids, assembly stabilizes the structure against biotic and abiotic destruction and transmissibility is based on their ability to function as templates. These peptide templates also bind generic and functionally diverse small molecules, much like nucleic acids bind histochemical dyes [86,87,88,89]. The prototypical dye Congo Red (CR) [88] binds end-to-end along the laminate groove of Ac-KLVFFAE-NH_2_ nanotube surfaces [64], and binds in a similar site-specific way to the HET-s prion of the filamentous fungus *Podospora anserine* [90,91]. These amyloid assemblies also bind oligoelectrolytes and poly(thiophene acetic acid) [92,93], much like RNA binding proteins [94], and other biological surfaces [95,96,97,98,99,100,101]. Experimental [102,103] and computational [104] evidence indicate that β-sheets can serve as templates for homochiral polymerization of N-carboxyanhydride activated amino acid monomers [103] and *in situ* activated α-amino acids [102]. Model self-replicating systems of β-sheet [105,106,107] peptides have been designed and the ability of β-sheets to template the polymerization of homochiral oligopeptides up to 30 residues long has been reviewed [108].

These activities may represent only a small fraction of the functions that provide the basis for the various amyloid diseases, and these capabilities have led to speculation about an amyloid world built on the potential of these surfaces to store and transfer information [109,110,111]. As impressive as these activities are, neither RNA nor amyloid alone achieve the functional diversity necessary for even the simplest cellular networks. The mutualism so evident in every step of information transmission in a cellular network suggests that life required both sides.

## 4. From Both Sides Now

**Figure 3:**
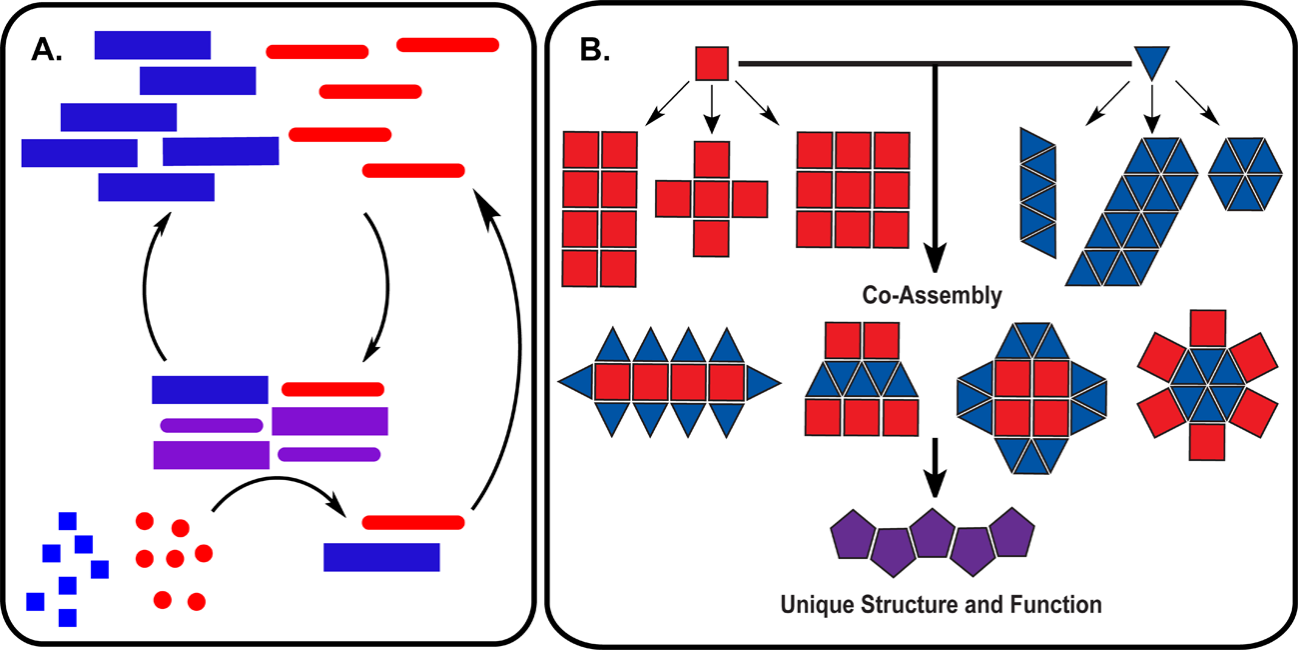
Autocatalysis and emergent functions from co-assemblies. (**A**) Co-assemblies formed from nucleic acid and peptide polymers catalyze the polymerization of additional polymers from monomer building blocks; (**B**) Co-assemblies derived from individual nucleic acid or peptide assemblies can significantly increase the structural diversity leading to emergent structures and functions.

Proteins and nucleic acids both store and process chemical information in cellular networks, but they do so interdependently; proteins are made from nucleic acid templates, and proteins read the templates. To the extent that there is a code or set of rules for such a mutualism may be revealed in the specific recognition of nucleic acids by RNA- and DNA-binding proteins and their elaborate structure–function relationship [112,113,114]. As mentioned above, the pinnacle may be the nucleic acid/peptide (NA/P) associations in the ribosome [9,115], but small nuclear ribonucleoprotein (snRNP) complexes and nucleases, including Ribonuclease P (RNaseP), provide simpler examples [5]. The woven intricacies of such co-assemblies are increasingly being defined [116,117] and have been reconstituted [9,118]. As rules for co-assembly emerge, co-evolutionary strategies become possible. Figure 3 outlines minimal functional capabilities that might be achieved with the co-assemblies. The nucleation of polymers (red and blue) might propagate as a template (purple) able to catalyze the independent production of more polymers (red and blue) in Figure 3A. Mutations in such a feedback system could allow for a minimal system capable of chemical evolution. This functional capability is of course dependent on the ability of these co-assemblies to transition to some unique functional form (mixture of squares and triangles, purple), as outlined in Figure 3B. While extant biology is a sophisticated network of efficiently functioning molecular partners, the many varied functions necessary for the existence of life, relies on the emergence of such co-assembling information networks capable of sustained growth in molecular order.

Data now exists for prion infectivity being altered by oligonucleotides [119,120,121,122,123], but little structural information is available. Similarly, *in vitro* guanine (dG)_16_ and cytosine (dC)_16_ hexadecamers, as well as their duplexes, associate with amphiphilic self-assembling peptides. The binding is pH dependent, occurs on a much faster timescale than for assembly of the neat peptide, and indeed gives rise to novel nucleic acid/peptide co-assemblies [124]. Hybridization of oligonucleotides containing sticky-ends can be catalyzed in the presence of self-assembling peptides, mimicking at least one integral step of replication [125]. And a five-nucleotide ribozyme produces multiple translational products, including the dipeptide FF [126]. In principle [127,128], when FF reaches the critical concentration for peptide self-assembly, the resulting peptide nanotube could selectively bind the ribozyme to create a dynamic system under feedback control.

Progress in this growing understanding of peptides and nucleic acids co-assembly may well depend on careful selection of systems, and the rich diversity of functional co-assemblies with existing biopolymers provides an exciting opportunity to define the basic mutualistic codes. In the simple assemblies outlined in Figure 3, the thermodynamic process of assembly would be physically coupled to polymerization of additional polymers from monomer building blocks, with the thermodynamic/kinetic tension manifested as feedback control. Elucidating the mechanisms of association, and the structural and functional diversity of the resulting complexes, will be necessary for a more general understanding of the chemical thresholds for early evolution of molecular mutualism. Simpler synthetic [129,130] and altered biopolymer [11,131,132] networks could then be extended to the dynamic processes of minimal native biopolymer co-assemblies [133,134,135,136].

## 5. Conclusions: Towards Molecular Mutualisms

The cooperative functioning of nucleic acids and peptides was recognized early with the designation of a central dogma decades ago, and it may well be that our attempts to simplify the system into metabolism-first, RNA-first, or amyloid-first has limited a more extensive exploration of mutualistic networks. Single biopolymer networks have contributed significantly to systems chemistry, but sidestepped the interdependence of metabolism and replication, analog and digital information processing, and the mutualism, so apparent in living systems, that is essential for the emergence of new functions [137]. The recognition of the importance of diverse, structurally complex, non-covalent assemblies in early evolution is certainly highlighted by their prevalance in biology [138,139,140,141], and routes to creating an ecology of biomolecules with sufficient functional dynamics to evolve chemically are now emerging. We may have reached the point where the creation of a mutualistic chemical ecology [142,143] can be used to inform the progressive growth of molecular information on Earth and understand the limits on chemical evolution broadly in our Universe [144,145,146]. As technology and methodologies to analyze complex networks improve, the next scientific discoveries may come from understanding harsh and extreme environments or even new “worlds” with fundamentally different systems of molecular networks [11,147,148]. Certainly we should now be able to define the limits on building to the complex networks we might call living.
